# Maternal 24-h movement patterns across pregnancy and postpartum: The LIFE-Moms consortium

**DOI:** 10.1016/j.pmedr.2024.102740

**Published:** 2024-04-26

**Authors:** Chelsea L. Kracht, Kimberly L. Drews, Emily W. Flanagan, Sarah K. Keadle, Dympna Gallagher, Linda Van Horn, Debra Haire-Joshu, Suzanne Phelan, Jeremy Pomeroy, Leanne M. Redman

**Affiliations:** a Pennington Biomedical Research Center, Baton Rouge, LA, USA; b University of Kansas Medical Center, Kansas City, KS 66160, USA; c California Polytechnic State University, San Luis Obispo, CA, USA; d Columbia University, New York City, NY, USA; e Northwestern University, Evanston, IL, USA; f Washinton University in St. Louis, St. Louis, MO, USA; g Marshfield Clinic Research Institute, Marshfield, WI, USA

**Keywords:** Sedentary time, Mother, Exercise, Perinatal, Movement, Sitting

## Abstract

**Objective:**

Time spent among the 24-h movement behaviors (physical activity [PA], sleep, sedentary behavior [SB]) in the perinatal period is important for maternal and child health. We described changes to 24-h movement behaviors and behavior guideline attainment during pregnancy and postpartum and identified correlates of behavior changes.

**Methods:**

This secondary data analysis included the standard of care group (*n* = 439) from the U.S.-based Lifestyle Interventions For Expectant Moms (LIFE-Moms) consortium, including persons with overweight and obesity. Wrist-worn accelerometry was used to measure movement behaviors early (9–15 weeks) and late (35–36 weeks) pregnancy, and ∼ 1-year postpartum. Sleep and moderate-to-vigorous PA (MVPA) were compared to adult and pregnancy-specific guidelines, respectively. SB was classified into quartiles. PA and SB context were quantified using questionnaires. Mixed models were used to examine changes in behaviors and guidelines and identify correlates.

**Results:**

Participants were 31.3 ± 3.5 years, 53.5 % were Black or Hispanic, and 45.1 % had overweight. Sleep duration decreased across time, but participants consistently met the guideline (range: 85.0–93.6 %). SB increased during pregnancy and decreased postpartum, while light PA and MVPA followed the inverse pattern. Participants met slightly fewer guidelines late pregnancy (1.2 ± 0.7 guidelines) but more postpartum (1.7 ± 0.8 guidelines) than early pregnancy (1.4 ± 0.8 guidelines). Black or Hispanic race/ethnicity, higher pregravid body mass index, and non-day work-shift (e.g., night-shift) were identified correlates of lower guideline adherence and varying PA and SB context.

**Conclusion:**

Perinatal interventions should consider strategies to prevent SB increase and sustain MVPA to promote guideline adherence.

## Highlights

• Sleep decreased from early pregnancy to postpartum, but within an acceptable range.
• Persons exhibited 40 more minutes of light physical activity postpartum.
• Number of children and work shift were important factors to exercise context based on surveys.

## 1 Introduction

Adequate amounts of physical activity (PA) and sleep, together with limited sedentary behavior (SB), are important for physical and mental health, especially for people during the perinatal period (Dipietro et al., 2019, Lu et al., 2021, Fazzi et al., 2017). However, pregnancy is well-characterized by variable sleep and a reduction in PA, with possible returns to pre-pregnancy levels around a year postpartum (Hesketh et al., 2018). Recognizing the exclusive and reciprocal properties of these behaviors, a 24-h activity cycle paradigm emerged to improve all three behaviors (Rosenberger et al., 2019, Tremblay et al., 2016). Perinatal movement behaviors are associated with child movement behaviors in some longitudinal cohort studies (Chen et al., 2019), but others report mixed results (Kracht et al., 2023). Even so, identifying correlates of 24-h movement behaviors in the perinatal period may help promote healthy behaviors for mother and child benefit.

Persons with overweight and obesity may be at risk for a greater decrease in PA and increased SB across gestation (Mitro et al., 2022, Daniali et al., 2023), and sleep disturbances (Lu et al., 2021). Past interventions to improve these behaviors in persons with overweight and obesity have reported limited effectiveness, as they mainly focus on improving a single behavior (Flannery et al., 2019). Understanding the types of PA and SB exhibited during the 24-h day may help discover opportunities to improve PA and replace SB. Using both devices (i.e., accelerometer) and questionnaires are recommended to help identify behavior patterns and context for future efforts (Gill et al., 2023), but have yet to be adapted in the perinatal period.

Examination of maternal movement behaviors has also been confined to primarily homogenous samples (mainly all Chinese or all white) (Chen et al., 2019, Kracht et al., 2023). Persons from communities of color and lower socioeconomic status face additional barriers to achieve a healthy lifestyle thus are a priority population for efforts to promote healthy amounts of PA, SB, and sleep (Taveras et al., 2010, Davison and Birch, 2001, Kindratt et al., 2023, Kracht et al., 2021). These barriers include more household crowding (Kracht et al., 2021), less maternal education (Kindratt et al., 2023) and employment (da Silva et al., 2018); which may also be influenced by perinatal factors, such as parity (Mitro et al., 2022, Badon et al., 2022). However, these home and individual-level correlates have not been explored across all three behaviors simultaneously and within a diverse sample.

The objective of the current study was to address these gaps by examining changes in device and questionnaire-based 24-h movement behaviors across the perinatal period in a diverse U.S.-based sample of persons with overweight and obesity (Aim 1). The second aim was to describe changes in 24-h movement guideline attainment during the perinatal period and identify correlates. We hypothesized that maternal race, characteristics of the home environment, parity, and employment would be related to meeting more guidelines.

## 2 Methods

This is a secondary analysis of the standard care group in the Lifestyle Interventions For Expectant Moms (LIFE-Moms) consortium, a group of behavioral clinical trials for adequate gestational weight gain (GWG) that occurred between 2012 and 2017 within multiple U.S sites (Clifton et al., 2016, Peaceman et al., 2018).

### 2.1 Participants

To be included in the study, pregnant people were 9–15 weeks gestation, had overweight or obesity (body mass index [BMI]: ≥25 kg/m^2^), and a confirmed singleton viable pregnancy. Persons were excluded for age <18 years, diabetes diagnosis, hemoglobin A1c ≥ 6.5 % prior to randomization, fetal anomaly, history of ≥3 consecutive miscarriages, and contraindications to beginning a diet and exercise-focused program (e.g., upcoming bariatric surgery). Recruitment occurred through various means, but most participants were recruited at their prenatal appointments (Clifton et al., 2016, Sutton et al., 2017, Gallagher et al., 2018, Van Horn et al., 2018, Cahill et al., 2018, Phelan et al., 2018).

Eligible participants were randomized to intervention or standard of care (i.e., control) groups within their respective sites. Individual trials were registered with the Clinical Trials Registry (NCT01545934, NCT01616147, NCT01771133, NCT01631747, NCT01768793, NCT01610752, NCT01812694). The LIFE-Moms Data Safety Monitoring Board and institutional review board for each site approved and monitored trial conduct and activities. Full study results are published elsewhere (Peaceman et al., 2018, Phelan et al., 2020). Five sites (California Polytechnical Institute-Brown University, Northwestern University, Pennington Biomedical Research Center, St. Lukes-Columbia University, and Washington University of St. Louis) contributing questionnaire, accelerometry, and anthropometry data from the standard of care (control) group were included.

### 2.2 Procedure

Signed informed consent was obtained prior to study procedures. Participants attended multiple visits during the perinatal period. The current analyses utilized weight, questionnaires, and accelerometry data obtained during early pregnancy (9–15 weeks gestation), late pregnancy (35–36 weeks gestation), and postpartum (1-year after birth). Persons also completed a demographic questionnaire and height (cm) was measured at the early pregnancy visit (Peaceman et al., 2018). Persons with baseline weights measured at 14-weeks and 15-weeks had 0.45 kg (1-pound) and 0.91 kg (2-pounds), respectively, subtracted for an estimate of their early pregnancy weight, as performed in other studies (Peaceman et al., 2018). Early pregnancy BMI was calculated using the standard formula (kg/m^2^).

### 2.3 24-h movement behaviors

Movement behavior duration was assessed using an Actigraph GT3X + accelerometer (Actigraph, LLC, Pensacola, FL) recorded at 50 Hz sampling rate, and placed on the wrist of the nondominant arm for improved adherence, and 24-h measurement (Rosenberger et al., 2019, Troiano et al., 2014). The accelerometer was worn for seven-days prior to each visit. Participants were instructed to wear the monitor continuously for the seven-days, including overnight and during water-based activities.

The GGIR algorithm was used to detect non-wear time then sustained activity based on angle variability to equate sleep (Migueles et al., 2019). Sleep hours were calculated from the time spent in “sleep” regardless of duration. Days with >10 h of awake wear and ≥20 h of total wear time (awake and sleep) were considered a valid day, and people with ≥1 valid day were included in analysis. GGIR version 1.11 was used to process PA metrics using raw accelerometer data (Migueles et al., 2019, Sabia et al., 2014, van Hees et al., 2013). The Euclidian Norm Minus One (ENMO), the vector magnitude of three axes minus 1 g (√[x^2^ + y^2^ + z^2^ ] − 1 g), was calculated and used to classify PA level. Activity was classified as inactive (0–39 mg), light ([LPA], 40 mg ≤ ENMO < 100 mg), and moderate-to-vigorous PA (MVPA, ENMO ≥ 100 mg) (Hildebrand et al., 2014). SB was operationalized as time spent inactive. Due to possible random wrist movement, bouts of ≥1 min where 80 % of the activity was classified as MVPA were used as performed by others (da Silva et al., Aug 2018). Variables were summarized for each participant for available days (minutes/day). Timepoints with valid PA and sleep data were included in the analysis.

PA and SB contexts were assessed using the Nurses Health Survey for PA questionnaire, a reliable and validated questionnaire (Wolf et al., 1994). Persons were asked based on the past year or since they completed the questionnaire, on average how much time they spent in various activities per week and number of days they exercised. There were ten categorical responses, increasing from minutes (0, 1–4, 5–19, 20–59) to hours (1, 1–1.5, 2–3, 4–6, 7–10, and 11 + . In a separate section of the questionnaire, participants were asked how many hours per week they spent sitting (options: away-from-home, watching television/ VCR/DVD [herein: screens], other), and standing/walking (options: at-work, at-home), with similar categorical responses. Responses were multiplied by appropriate conversion factors to calculate minutes/week. The PA portion of the questionnaire was only administered at three sites, but the SB portion was administered at all five sites due to varying site protocols.

### 2.4 24-h movement behavior guidelines

The World Health Organization (WHO) PA guideline recommendation for pregnancy is ≥150 min/week of moderate intensity PA on ≥3/days (Bull et al., 2020), aligning with the PA Guidelines for Americans pregnancy recommendation (Piercy et al., 2018). This guideline was then operationalized as averaging ≥22 min/day of 1-minute bouts of MVPA (as described previously) across available days as performed by others (Kwon et al., 2022), approximately 150-min/7-days.

The current WHO guidelines focus on replacing time spent sedentary with other activity intensities in pregnancy and postpartum periods (Bull et al., 2020), and does not have a quantitative SB guideline. SB duration was divided into quartiles, and the lowest quartile (least time spent sedentary) was operationalized as meeting the guideline based on prior work (Ekelund et al., 2019, Lee et al., 2018). The lowest quartile from the sample at early pregnancy (<9.5 h/day) was applied to other periods for consistency.

The average amount of daily sleep was compared to the National Sleep Foundation guideline for sleep (7–9 h/day) (Hirshkowitz et al., 2015). The number of guidelines met was the sum of guidelines met (PA, SB, and sleep) per timepoint (range: 0–3).

### 2.5 Correlates

Correlates were assessed early pregnancy via questionnaires. Individual factors included age, parity (range: 0–4), race/ethnicity (Non-Hispanic White, Non-Hispanic Black/African American, Hispanic, and other/mixed race), household income (<$25,000/year, $25,000–75,000/year, >$75,000/year), maternal employment (unemployed, employed day-shifts, employed-other shifts [afternoon, night, split, irregular, or rotating]), and recruitment site. Home-based factors included number of televisions in the home (1, 2, 3+) and living situation as a proxy for household crowding (owns home, rents, or lives with parent or other adults).

### 2.6 Statistical analysis

For aim 1, a series of generalized linear mixed models were conducted to evaluate changes in individual behaviors (MVPA, LPA, SB, and sleep), PA types, days of exercise, and SB contexts across early to late pregnancy, and early pregnancy to postpartum. Models included time as a random variable, and fixed covariates of age, parity, early pregnancy BMI, race, income, employment, living situation, televisions, and site, and a model-based covariance matrix of fixed effects (Kenward and Roger, 1997). For aim 2, to examine changes in guideline attainment and identify correlates, generalized linear mixed models were used to evaluate changes in number of guidelines (range: 0–3) across the same time frames and adjusted models from Aim 1. In all aims, significant correlates were the same when conducted as early pregnancy and postpartum comparisons, thus correlate estimates from early to late pregnancy were presented for all analyses. A sensitivity analysis was conducted with people who contributed ≥ 3 days of valid PA and sleep for accelerometry measures (*n* = 419) (da Silva et al., 2021), and results were sustained. All analysis were conducted in using SAS 9.4 (Cary, N.C.), and statistical significance was set at *p* < 0.05.

## 3 Results

Amongst the 481 persons in the control group, 454 had ≥ 1 day of PA and sleep for at least one time point. After removal of outliers (*n* = 15), 439 people were included in analysis for Aim 1 (Table 1). Those not included in analysis (*n* = 42), were more likely to have a lower income, class I obesity, and rent their home compared to those included (Supplementary Table 1). Included persons were 31.3 ± 3.5 years of age, 53.5 % were Black or Hispanic, and majority had overweight during early pregnancy (45.1 %). On average, people contributed around 4–5 valid days of PA (early: 5.0 ± 1.5, late: 4.8 ± 1.5, postpartum: 4.0 ± 1.5), and sleep (early: 5.6 ± 2.1, late: 5.0 ± 2.2, postpartum: 4.9 ± 2.9) at each time point.

**Table 1:** Characteristics of included individuals with overweight and obesity in a U.S.-based cohort conducted in 2012–2017 (n = 439).

		n (%)	Mean ± SD
Age			31.3 ± 3.5
Race/ethnicity			
	White	176 (40.1)	
	Black	148 (33.7)	
	Hispanic	87 (19.8)	
	Other	28 (6.7)	
Income			
	<25,000 USD	148 (33.8)	
	25–75,000 USD	115 (26.3)	
	>75,000 USD	174 (39.8)	
Parity			1.0 ± 1.0
Early pregnancy BMI			31.4 ± 4.7
Early pregnancy Obesity Class			
	Overweight (BMI: 25–29.9)	198 (45.1)	
	Obesity Class I (30.0–34.9)	140 (31.9)	
	Obesity Class II (35.0–39.9)	70 (16.0)	
	Obesity Class III (40.0 or greater)	31 (7.0)	
Early pregnancy Employment			
	Employed (day shift)	227 (51.7)	
	Employed (other shift)	99 (22.5)	
	Not employed	113 (25.7)	
Early pregnancy living situation			
	Own house/condo	159 (36.3)	
	Rent	225 (51.3)	
	Live in home of parents or other adults	54 (12.3)	
TVs in the home			
	1 TV	119 (27.1)	
	2 TVs	150 (34.2)	
	3 + TVs	170 (38.7)	

BMI = pre-pregnancy BMI; GWG = gestational weight gain; MVPA = moderate-to-vigorous physical activity; Other race/ethnicity was included as a fourth response option and included those who did not identify as White, Black, or Hispanic.

### 3.1 24-h movement behaviors

**Fig. 1:**
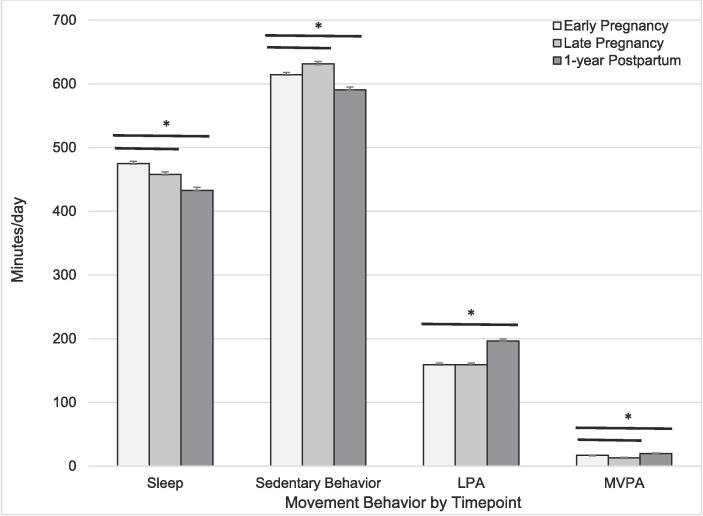
Device-based Maternal Movement Behaviors by Timepoint in persons with Overweight and Obesity in a U.S.-based cohort conducted in 2012–2017 (n = 439). Figure Legend: *Population includes those with at least ≥ 1 day of valid sleep and physical activity from accelerometry, estimates shown are least square means*; *Assessed using generalized linear regression without adjustment, p < 0.05; MVPA = moderate-to-vigorous physical activity; LPA = light physical activity; white gray = early pregnancy; light gray = late pregnancy; dark gray = 1-yr postpartum, dark bars indicate significant difference between time points.*

In unadjusted models, all three movement behaviors changed across time (*p* < 0.05, Fig. 1). Compared to early pregnancy (17.1 ± 0.5 min/day), MVPA had a slight decline in late pregnancy (13.3 ± 0.5 min/day) and returned to early pregnancy levels by 1-year postpartum (19.7 ± 0.7 min/day). SB followed the converse path; it increased from early to late pregnancy (10.3 ± 0.1 vs. 10.5 ± 0.6 h/day, respectively) and decreased postpartum (9.8 ± 0.7 h/day). LPA did not change across pregnancy (early: 159.3 ± 2.4 min/day, late: 159.9 ± 2.7 min/day, *p* = 0.77). LPA was ∼40 min/day higher postpartum (195.8 ± 3.1 min/day) compared to early pregnancy. Sleep declined late pregnancy (7.6 ± 0.5 h/day) and postpartum (7.2 ± 0.1 h/day) compared to early pregnancy (7.9 ± 0.5 h/day).

In adjusted models, higher parity was negatively related to SB (*β* ± *SE*:-0.16 ± 0.05 h/day) and sleep (*β* ± *SE*:-0.17 ± 0.05 h/day), translating into ∼ 10 less minutes/day of SB and sleep per additional child (p < 0.05 for both, Supplementary Table 2). Higher parity was positively related to LPA, with 12.2 ± 2.3 more minutes/day per additional child (*p* = 0.001). Persons with middle (*β* ± *SE*:-18.8 ± 6.6 min/day) and low income (*β* ± *SE*:-19.9 ± 8.5 min/day) had less LPA compared to those with high income (*p* < 0.05 for both). People who were unemployed had less MVPA (*β* ± *SE*:3.01 ± 1.18 min/day, *p* = 0.01) but more sleep (*β* ± *SE*:0.35 ± 0.13 h/day, *p* = 0.01) compared to those who worked the day-shift.

### 3.2 Physical activity and sedentary behavior context

Unadjusted models revealed aerobic activity, arm weights, bicycling, jogging, and leg weights decreased from early to late pregnancy (*p* < 0.05, Table 2), with persons reporting around 20 min/week less in each of these activities. Persons continued to spend less time in arm weights, bicycling, leg weights, and yoga at 1-year postpartum compared to early pregnancy (*p* < 0.05). Persons also spent slightly fewer days engaging in exercise during late pregnancy (2.4 ± 0.1 days) compared to early pregnancy (2.7 ± 0.1, *p* = 0.04), but not postpartum (2.6 ± 0.1, *p* = 0.77). Time spent standing outside-of-the-home decreased between early and late pregnancy (*p* = 0.002). Persons spent less time sitting in all contexts when postpartum is compared to early pregnancy (*p* < 0.05). In contrast, persons increased their standing at-home time from early pregnancy (37.3 ± 2.2 h/week) to postpartum (51.5 ± 2.8 h/week, *p* < 0.05).

**Table 2:** Unadjusted physical activity and sedentary behavior context across pregnancy and postpartum in person with overweight and obesity in a U.S.-based cohort conducted in 2012–2017 (*n* = 439).

	**Early Pregnancy** **(*n* = 303)**	**Late Pregnancy (*n* = 158)**		**1-year Postpartum** **(*n* = 150)**	***p*-value**

**Physical Activity Context (minutes/week)**					

Aerobic activity	36.8 ± 4.0	11.2 ± 5.2	0.001*	25.7 ± 5.9	0.07
Arm weights	31.3 ± 3.3	10.1 ± 4.1	0.001*	21.5 ± 4.3	0.01*
Bicycling	25.9 ± 4.0	3.2 ± 5.7	0.001*	8.7 ± 5.9	0.009*
Jogging	29.0 ± 3.5	10.6 ± 4.9	0.001*	23.3 ± 5.2	0.31
Leg weights	28.0 ± 3.2	5.0 ± 4.4	0.001*	13.9 ± 4.5	0.002*
Racket sports	2.6 ± 0.9	0.5 ± 1.1	0.18	0.3 ± 1.2	0.16
Running	14.1 ± 3.1	6.5 ± 4.2	0.14	14.2 ± 4.3	0.99
Swimming	9.3 ± 2.5	7.0 ± 2.7	0.12	2.1 ± 3.0	0.05
Vigorous activities (other)	15.5 ± 2.2	13.8 ± 3.0	0.61	20.2 ± 3.5	0.26
Walking	136.9 ± 9.2	135.2 ± 12.3	0.90	138.1 ± 12.7	0.93
Yoga	34.3 ± 3.5	30.7 ± 4.4	0.38	22.2 ± 4.5	0.001*
Exercise days/week	2.7 ± 0.1	2.4 ± 0.1	0.04*	2.6 ± 0.1	0.77

	**Early Pregnancy** **(*n* = 439)**	**Late Pregnancy (*n* = 375)**		**1-year Postpartum** **(*n* = 351)**	

**Sedentary Behavior Context (hours/week)**					

Sitting − Away from home	16.7 ± 0.8	16.4 ± 0.8	0.73	14.5 ± 0.9	0.02*
Sitting − TV/Screens	10.8 ± 0.6	11.4 ± 0.6	0.43	8.7 ± 0.6	0.004*
Sitting − Other home	8.6 ± 0.5	8.5 ± 0.5	0.84	7.0 ± 0.5	0.008*
Standing- Away from home	16.8 ± 0.8	14.2 ± 0.9	0.002*	18.5 ± 0.9	0.14
Standing − Home	36.5 ± 2.2	36.1 ± 2.4	0.90	51.7 ± 2.8	0.001*

^Assessed using generalized linear mixed models adjusted for time, Least Square Means ± standard error is presented; p-value is comparison of early to late pregnancy; p < 0.05*.

In adjusted models by PA or SB context (Supplementary Table 3–6), age was positively related to five PA types; but negatively related to other sitting activities at-home (p < 0.05 for all). Higher parity was related to less bicycling and yoga (*p* < 0.05 for both). Pre-pregnancy BMI was negatively related to jogging and running but positively related to sitting for television/screens (*p* < 0.05 for all). Relative to non-Hispanic White counterparts, persons who were non-Hispanic Black engaged in less of three PA types, while persons who were Hispanic engaged in fewer exercise days and SB contexts (p < 0.05 for all). Compared to persons with high income, persons with middle or low income spent less time in three PA types and sitting away-from-home (p < 0.05 for all). Compared to persons with a day shift, persons employed on non-day shifts engaged in fewer exercise days and differed across SB contexts (p < 0.05 for all). Persons who rented their homes or had 2 TVs in their home demonstrated different engagement in only one PA type compared to their counterparts (p < 0.05 for both).

### 3.3 Guideline attainment

**Fig. 2:**
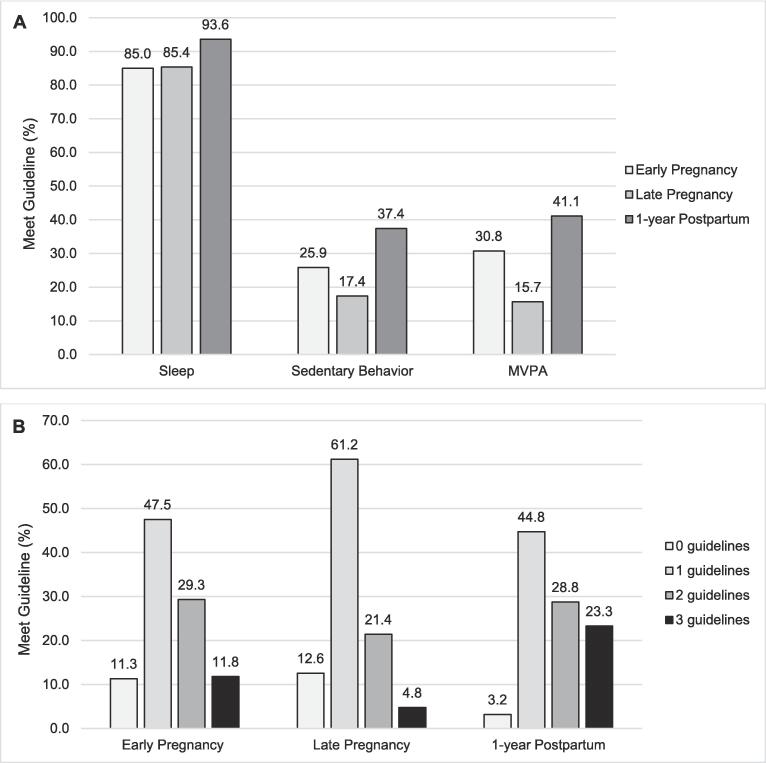
Device-based Movement Behavior Guideline attainment across early pregnancy, late pregnancy, and postpartum in persons with overweight and obesity in a U.S.-based cohort conducted in 2012–2017 (*n* = 439) Panel A: Movement Behavior Guidelines; Panel B: Number of Guidelines Legend: *Panel A: white gray = early pregnancy; light gray = late pregnancy; dark gray = 1-yr postpartum; MVPA = moderate-to-vigorous physical activity; Panel B: white = 0 guidelines, light gray = 1 guideline, dark gray = 2 guidelines, black = 3 guidelines. Population includes those with at least ≥ 1 day of valid sleep and physical activity from accelerometry; Early pregnancy includes 406 mothers, late pregnancy includes 294 mothers, and postpartum includes 219 mothers.*

MVPA and SB guideline attainment was lowest late pregnancy compared to early pregnancy and postpartum, indicating low MVPA and more SB (Fig. 2). Persons consistently met the sleep guideline across the prenatal period (range: 85.0–93.6 %). The proportion of those who met all three guidelines was highest at 1-year postpartum, compared to late and early pregnancy. Compared to early pregnancy (1.4 ± 0.03), persons met slightly fewer guidelines late pregnancy (1.2 ± 0.04, *p* < 0.001) but slightly more guidelines postpartum (1.7 ± 0.05, *p* < 0.001). In adjusted models, early pregnancy BMI was negatively related to total guidelines (*β* ± *SE*: −0.01 ± 0.007, *p* = 0.04). Persons who were non-Hispanic Black met fewer guidelines compared to persons who were non-Hispanic White (*β* ± *SE*: −0.29 ± 0.13, *p* = 0.04**,** Supplementary Table 5).

## 4 Discussion

This study sought to describe changes in 24-h movement behaviors and guideline attainment across the perinatal period and identify correlates of behaviors. Each 24-h movement behavior followed a unique pattern, with some having slight changes (MVPA and SB), and others having a steady decline (sleep) or steady increase (LPA). Persons attained slightly more guidelines postpartum compared to early pregnancy, but many only met the sleep guideline. People with higher BMI and who were Black achieved fewer guidelines, while parity, living situation, and employment status were important for individual behaviors and context. Persons with overweight and obesity may already have adequate sleep duration but benefit from strategies to promote home and individual PA options to be active and sit less to promote recommended 24-h movement behaviors.

MVPA reductions are well described across pregnancy (Mitro et al., 2022), but it was unclear if this time was re-allocated to SB or if it became LPA (Hesketh et al., 2018, Badon et al., 2022). Data from this continental U.S. cohort supports that MVPA was re-allocated to SB as exercise days decreased but LPA stayed the same. Persons continued their walking and yoga time late pregnancy, which may be LPA based on their lower relative intensity (Ainsworth et al., 2011). It is likely MVPA time was reallocated to time spent sitting watching television as this was the only SB with an increase late pregnancy, albeit it was not statistically significant. The alignment of results encourages including both device- and questionnaire-based measures to provide context and intensity.

People with overweight and obesity met slightly more 24-h movement behavior guidelines postpartum. This is the result of resuming MVPA, increasing LPA, decreasing SB, and slight change in sleep relative to early pregnancy. This cohort exhibited similar adherence to all three guidelines early pregnancy (11.8 %) as general adult and child populations (7 %) (Rollo et al., 2022, Tapia-Serrano et al., 2022), but was higher 1-year postpartum (23.0 %). Persons with overweight and obesity may have sleep disturbances (Lu et al., 2021), but the duration may be within an acceptable range a year after birth. Sleep and time spent sitting at-home (TV/screens and other) was likely reallocated to standing/walking at-home postpartum. Whereas in late pregnancy MVPA decreases and SB increases (Mitro et al., 2022, Daniali et al., 2023), thus impacting two of the three guidelines and resulting in lower guideline attainment. The PA guideline is applicable across trimesters, though others have advocated for trimester-specific guidance (Hayman et al., 2023). These results suggest focusing efforts on increasing or sustaining MVPA across pregnancy and focusing on reducing SB during this time for higher guideline attainment.

Our hypotheses were partially supported; as guideline attainment differed by pregravid BMI and race/ethnicity, though other correlates (e.g., employment) identified were only important for behavior context (Mitro et al., 2022, Badon et al., 2022). Black children and adults meet fewer overall guidelines compared to White counterparts (Kindratt et al., 2023, Kracht et al., 2019), which may also apply to people entering pregnancy. Racist structures, institutions and people may be contributing to this trend. Black people face more barriers to healthy sleep and activity, including stress from systemic racism and living in neighborhoods with less access to safe areas for PA (Lofton et al., 2023). Higher maternal BMI was also associated with multiple types of physical activities (e.g., less running and jogging), strengthening a focus on other modalities that achieve MVPA (e.g., dancing or aerobics classes) and possibly utilizing the higher television time to stream these exercises. Home environment metrics (i.e., living situation and number of TVs) were not related to guidelines but were related to a few individual behaviors. Other metrics of home environment, such as household chaos (Kracht et al., 2021), may better encapsulate the impact of the home environment on behavior rather than physical environment characteristics. Parity was related to individual behaviors in mixed directions, suggesting support for both nulliparous and multiparous people to increase guideline attainment. Though not related to guidelines, a consistent factor for individual behaviors and context was employment and shift (i.e., day vs. non-day shift), building upon existing findings for overall employment during pregnancy (da Silva et al., 2018) and education (Kindratt et al., 2023). Shift workers may be more sedentary overall and exhibit less vigorous PA on off-days than day shift counter parts (Feng et al., 2021). The current study results suggest number of PA days and type of activity may play a role in PA obtained during the perinatal period, and be an area for intervention during pregnancy. Multi-level approaches are required to provide options to support different day and night schedules so people can continue activity and reduce SB through pregnancy.

The rigorous and harmonized measures of the LIFE-Moms consortium, and use of device-based and questionnaire assessment of PA, SB, and sleep is an extension of past research through the inclusion of a diverse population of the U.S., and assessment of all three 24-h movement behaviors (Clifton et al., 2016, Peaceman et al., 2018). Participation and interest of participants in a randomized control trial to reduce GWG may not generalize to those who did not participate. We were unable to separate moderate and vigorous PA in the GGIR algorithm, though pregnancy recommendations are specific to MPA so our guideline estimates may be conservative. Another guideline consideration is that our SB guideline was based on this population’s lower quartile (9.5 h/day), which demonstrates deleterious health benefits in adults (Ekelund et al., 2019), but may not generalize to other perinatal populations. Further, the LIFE-Moms cohort may not generalize to other countries with differing maternity leave policies which may better support maternal health in late pregnancy and throughout the first year post-birth (Cardenas et al., 2021). Our analysis only considered total time spent in each behavior, and other metrics of sleep (e.g., quality and efficiency) and SB (e.g., bouts and bout length) may further elucidate the patterns and changes across the perinatal period. Moreover, wrist-worn accelerometry is an improved measure of sleep for 24-h assessment, but it is a suboptimal measure of SB (Rosenberger et al., 2019, Troiano et al., 2014). This study did not collect time-use diaries, thus it is not possible to assess exact trade-offs in behaviors during the three observation periods. We acknowledge that another limitation is having one postpartum timepoint (1-year). Within this sample there was also a decrease in attendance, as one site did not include accelerometry postpartum and others were lost to follow up. Indeed, sleep may differ across the first year postpartum especially in the first 3-months with around the clock newborn care (Richter et al., 2019). Longitudinal examination of all 24-h movement behaviors across other timepoints postpartum may improve upon these results (Hesketh et al., 2018).

There are three clear future directions from these findings. First, it is important to extend our understanding of time allocation from one movement behavior to another on maternal and infant health outcomes. This may be achieved through a compositional data analysis method, which has been applied to other populations and linked to health outcomes (Janssen et al., 2020) but not the perinatal period (Sandborg et al., 2022, Kracht and Redman, Sep 2023). A second consideration is providing actionable options to continue MVPA, like vigorous intermittent lifestyle PA, or short bouts of vigorous activity, which demonstrate health benefit (Stamatakis et al., 2021), and may be feasible amongst busy mothers at 12-months postpartum. Third, MVPA in late pregnancy will likely not mirror early pregnancy MVPA due to pregnancy-related cardiorespiratory adaptations and studies are needed to identify exercises applicable across gestation (Birnbaumer et al., 2020).

The current study contributes to the evidence that people with overweight and obesity may improve their 24-h movement profile postpartum but require additional support early in gestation. Home-based and individual options for PA and less SB should consider how a person spends their day and night. Supporting healthy amounts of all three behaviors during pregnancy may improve 24-h movement guideline adherence for maternal and child health.

## CRediT authorship contribution statement

**Chelsea L. Kracht:** Writing – review & editing, Writing – original draft, Visualization, Formal analysis, Conceptualization. **Kimberly L. Drews:** Writing – review & editing, Project administration, Methodology, Investigation, Funding acquisition, Formal analysis, Data curation. **Emily W. Flanagan:** Writing – review & editing, Conceptualization. **Sarah K. Keadle:** Writing – review & editing, Data curation, Conceptualization. **Dympna Gallagher:** Writing – review & editing, Project administration, Methodology, Investigation, Funding acquisition. **Linda Van Horn:** Writing – review & editing, Project administration, Methodology, Investigation, Funding acquisition. **Debra Haire-Joshu:** Writing – review & editing, Project administration, Methodology, Investigation, Funding acquisition. **Suzanne Phelan:** Writing – review & editing, Project administration, Methodology, Investigation, Funding acquisition. **Jeremy Pomeroy:** Writing – review & editing, Project administration, Methodology, Investigation, Funding acquisition, Data curation. **Leanne M. Redman:** Writing – review & editing, Project administration, Methodology, Investigation, Funding acquisition, Conceptualization.

## Declaration of competing interest

The authors declare that they have no known competing financial interests or personal relationships that could have appeared to influence the work reported in this paper.

## Data Availability

Data will be made available on request.
