# Supplementary material for: Maternal 24-h movement patterns across pregnancy and postpartum: The LIFE-Moms consortium
Source: Prev Med Rep. 2024 Apr 26;42:102740. doi: 10.1016/j.pmedr.2024.102740 (PMC11068928; doi:10.1016/j.pmedr.2024.102740)
Supplement: Supplementary Data 1 [file mmc1.docx]

| Supplementary Table 1. Characteristics between included and excluded participants with overweight and obesity in a U.S.-based cohort conducted in 2012-2017 (n=481) | | | | | | |
| --- | --- | --- | --- | --- | --- | --- |
|  | | Included  (n=439) | | Excluded  (n=42) | |  |
|  | | n (%) | Mean ± SD | n (%) | Mean ± SD | p-value |
| Age |  |  | 31.3 ± 3.5 |  | 31.7 ± 3.2 | 0.54 |
| Race |  |  |  |  |  | 0.43 |
|  | White | 176 (40.1) |  | 12 (28.6) |  |  |
|  | Black | 148 (33.7) |  | 17 (40.5) |  |  |
|  | Hispanic | 87 (19.8) |  | 11 (26.2) |  |  |
|  | Other | 28 (6.7) |  | 2 (4.7) |  |  |
| Income | |  |  |  |  | 0.03* |
|  | <25,000 USD | 148 (33.8) |  | 23 (54.8) |  |  |
|  | 25-75,000 USD | 115 (26.3) |  | 8 (19.0) |  |  |
|  | >75,000 USD | 174 (39.8) |  | 11 (26.2) |  |  |
| Parity | |  | 1.0 ± 1.0 |  | 1.2 ± 1.2 | 0.08 |
| Early pregnancy BMI | |  | 31.4 ± 4.7 |  | 32.3 ± 3.9 | 0.27 |
| Early pregnancy Obesity Class | |  |  |  |  | 0.02* |
|  | Overweight (BMI: 25-29.9) | 198 (45.1) |  | 11 (26.2) |  |  |
|  | Obesity Class I (30.0-34.9) | 140 (31.9) |  | 23 (54.8) |  |  |
|  | Obesity Class II (35.0-39.9) | 70 (16.0) |  | 6 (14.3) |  |  |
|  | Obesity Class III (40.0 or greater) | 31 (7.0) |  | 2 (4.7) |  |  |
| Early pregnancy Employment | |  |  |  |  | 0.05 |
|  | Employed (day shift) | 227 (51.7) |  | 15 (35.7) |  |  |
|  | Employed (other shift) | 99 (22.5) |  | 16 (38.1) |  |  |
|  | Not employed | 113 (52.7) |  | 11 (26.2) |  |  |
| Early pregnancy living situation | |  |  |  |  | 0.03* |
|  | Own house/condo | 159 (36.3) |  | 8 (19.0) |  |  |
|  | Rent | 225 (51.3) |  | 30 (71.4) |  |  |
|  | Live in home of parents or other adults | 54 (12.3) |  | 4 (9.5) |  |  |
| TVs in the home | |  |  |  |  | 0.14 |
|  | 1 TV | 119 (27.1) |  | 9 (21.4) |  |  |
|  | 2 TVs | 150 (34.2) |  | 21 (50.0) |  |  |
|  | 3+ TVs | 170 (38.7) |  | 12 (28.5) |  |  |
| *^Assessed using One-Way ANOVA or chi-square/fisher test; p<0.05*; BMI = pre-pregnancy BMI; GWG= gestational weight gain; MVPA= moderate-to-vigorous physical activity.* | | | | | | |

| Supplementary Table 2. Associations between correlates and maternal device-based movement behaviors across early and late pregnancy in a U.S.-based cohort conducted in 2012-2017 | | | | | | | | | |
| --- | --- | --- | --- | --- | --- | --- | --- | --- | --- |
|  |  | Sleep | | Sedentary Behavior | | LPA | | MVPA | |
|  |  | hours/day | | hours/day | | minutes/day | | minutes/day | |
|  |  | *β*±*SE* | p-value | *β*±*SE* | p-value | *β*±*SE* | p-value | *β*±*SE* | p-value |
| Time |  | -0.29 ± 0.07 | <.0001* | 0.27 ± 0.06 | <.0001* | 0.36 ± 2.49 | 0.88 | -3.81 ± 0.52 | <.0001* |
| Age |  | -0.0002 ± 0.004 | 0.94 | -0.001 ± 0.003 | 0.76 | 0.09 ± 0.16 | 0.56 | 0.016 ± 0.03 | 0.65 |
| Race |  |  |  |  |  |  |  |  |  |
|  | White | Ref |  | Ref |  | Ref |  | Ref |  |
|  | Hispanic | -0.08 ± 0.15 | 0.59 | -0.04 ± 0.14 | 0.78 | -0.93 ± 6.24 | 0.88 | 0.34 ± 1.35 | 0.79 |
|  | Black | 0.10 ± 0.19 | 0.60 | 0.32 ± 0.19 | 0.08 | -6.15 ± 7.94 | 0.43 | -3.00 ± 1.72 | 0.08 |
|  | Other | -0.21 ± 0.23 | 0.34 | 0.29 ± 0.22 | 0.18 | 0.49 ± 9.23 | 0.95 | -3.40 ± 2.00 | 0.08 |
|  | White |  |  |  |  |  |  |  |  |
| Income | |  |  |  |  |  |  |  |  |
|  | >75,000 USD | Ref |  | Ref |  | Ref |  | Ref |  |
|  | 25-75,000 USD | -0.27 ± 0.15 | 0.07 | -0.27 ± 0.14 | 0.06 | 18.84 ± 6.16 | 0.002* | -0.0001 ± 1.33 | 0.99 |
|  | <25,000 USD | -0.35± 0.21 | 0.09 | -0.24 ± 0.20 | 0.22 | 19.93 ± 8.54 | 0.02* | 1.29 ± 1.85 | 0.48 |
| Parity |  | -0.17± 0.05 | 0.003* | -0.16 ± 0.05 | 0.003* | 12.25 ± 2.30 | <.0001* | -0.11 ± 0.50 | 0.82 |
| Early pregnancy BMI | | -0.005± 0.01 | 0.63 | 0.006 ± 0.01 | 0.52 | -0.73 ± 0.43 | 0.09 | -0.16 ± 0.09 | 0.08 |
| Early pregnancy Employment | |  |  |  |  |  |  |  |  |
|  | Employed (day shift) | Ref |  | Ref |  | Ref |  | Ref |  |
|  | Employed (other shift) | -0.01 ± 0.13 | 0.93 | -0.23 ± 0.13 | 0.07 | 7.16 ± 5.57 | 0.19 | -1.89 ± 1.20 | 0.11 |
|  | Not employed | 0.35 ± 0.13 | 0.01* | -0.01 ± 0.13 | 0.91 | -1.23 ± 5.45 | 0.82 | -3.01 ± 1.18 | 0.01* |
| Early pregnancy living situation | |  |  |  |  |  |  |  |  |
|  | Own house/condo | Ref |  | Ref |  | Ref |  | Ref |  |
|  | Rent | 0.22 ± 0.13 | 0.10 | -0.05 ± 0.12 | 0.69 | -6.51 ± 5.36 | 0.22 | 1.54 ± 1.75 | 0.37 |
|  | Live in home of parents or other adults | 0.04 ± 0.20 | 0.83 | 0.28 ± 0.19 | 0.14 | -8.49 ± 8.06 | 0.29 | 1.27 ± 1.47 | 0.38 |
| TVs in the home | |  |  |  |  |  |  |  |  |
|  | 1 TV | Ref |  | Ref |  | Ref |  | Ref |  |
|  | 2 TVs | 0.21 ±0.13 | 0.11 | -0.22 ± 0.12 | 0.08 | 6.98 ± 5.39 | 0.19 | 1.29 ± 1.21 | 0.28 |
|  | 3+ TVs | 0.11 ± 0.13 | 0.42 | -0.02 ± 0.13 | 0.84 | 1.77 ± 5.60 | 0.75 | 1.00 ± 1.08 | 0.35 |
| Site |  |  | 0.003* |  | 0.21 |  | 0.78 |  | 0.003* |
| *^Assessed using generalized mixed models; Time represents difference between late pregnancy and early pregnancy; Site p-value is from type III test of fixed effects; LPA = light physical activity; MVPA = moderate-to-vigorous physical activity; Ref = referent; *p<0.05* | | | | | | | | | |

| Supplementary Table 3. Associations between correlates and selected physical activity contexts across early and late pregnancy in a U.S.-based cohort conducted in 2012-2017 | | | | | | | | | |
| --- | --- | --- | --- | --- | --- | --- | --- | --- | --- |
|  |  | Aerobic Activity | | Arm weights | | Bicycling | | Jogging | |
|  |  | minutes/week | | minutes/week | | minutes/week | | minutes/week | |
|  |  | *β*±*SE* | p-value | *β*±*SE* | p-value | *β*±*SE* | p-value | *β*±*SE* | p-value |
| Time |  | -14.82 ± 2.63 | <.0001* | -13.77 ± 2.13 | <.0001* | -9.29 ± 2.33 | 0.0001* | -14.39 ± 2.54 | 0.001* |
| Age |  | 0.02 ± 0.10 | 0.80 | 0.24 ± 0.10 | 0.02* | 0.02 ± 0.08 | 0.75 | 0.27 ± 0.09 | 0.003* |
| Race |  |  |  |  |  |  |  |  |  |
|  | White | Ref |  | Ref |  | Ref |  | Ref |  |
|  | Black | 2.44 ± 4.94 | 0.62 | 0.87 ± 4.79 | 0.85 | -0.64 ± 3.62 | 0.85 | -1.43 ± 4.28 | 0.73 |
|  | Hispanic | 1.90 ± 3.57 | 0.59 | 0.46 ± 3.43 | 0.89 | -1.71 ± 2.59 | 0.51 | -1.73 ± 3.10 | 0.57 |
|  | Other | 2.58 ± 5.78 | 0.65 | -1.51 ± 5.50 | 0.78 | -3.55 ± 4.14 | 0.39 | 4.95 ± 4.95 | 0.31 |
| Income | |  |  |  |  |  |  |  |  |
|  | >75,000 USD | Ref |  | Ref |  | Ref |  | Ref |  |
|  | 25-75,000 USD | -7.09 ± 3.63 | 0.05 | -8.31 ± 3.47 | 0.01* | -3.43 ± 2.62 | 0.19 | -4.46 ± 3.11 | 0.15 |
|  | <25,000 USD | -7.14 ± 5.27 | 0.17 | -11.61 ± 5.11 | 0.02* | 3.08 ± 3.85 | 0.42 | -3.08 ± 4.57 | 0.50 |
| Early pregnancy BMI | | -0.32 ± 0.30 | 0.27 | -0.10 ± 0.29 | 0.72 | -0.003 ± 0.22 | 0.98 | -0.73 ± 0.26 | 0.005* |
| Parity |  | -1.77 ± 1.68 | 0.29 | -1.93 ± 1.62 | 0.23 | -2.62 ± 1.24 | 0.03* | -1.65 ± 1.47 | 0.73 |
| Early pregnancy Employment | |  |  |  |  |  |  |  |  |
|  | Employed (day shift) | Ref |  | Ref |  | Ref |  | Ref |  |
|  | Employed (other shift) | -4.40 ± 3.79 | 0.24 | -0.58 ± 3.67 | 0.87 | -4.12 ± 2.73 | 0.13 | 1.09 ± 3.25 | 0.73 |
|  | Not employed | -3.05 ± 3.92 | 0.43 | -1.39 ± 3.79 | 0.71 | 2.39 ± 2.89 | 0.40 | 3.21 ± 3.41 | 0.34 |
| Early pregnancy living situation | |  |  |  |  |  |  |  |  |
|  | Own house/condo | Ref |  | Ref |  | Ref |  | Ref |  |
|  | Rent | -3.23 ± 3.35 | 0.32 | 1.46 ± 3.32 | 0.66 | -1.91 ± 2.45 | 0.43 | 2.43 ± 2.89 | 0.40 |
|  | Live in home of parents or other adults | -4.35 ± 5.97 | 0.46 | 2.26 ± 3.77 | 0.54 | -7.81 ± 4.39 | 0.07 | 5.75 ± 5.35 | 0.28 |
| TVs in the home | |  |  |  |  |  |  |  |  |
|  | 1 TV | Ref |  | Ref |  | Ref |  | Ref |  |
|  | 2 TVs | 0.21 ± 3.45 | 0.94 | -3.01 ± 3.22 | 0.35 | -0.60 ± 2.52 | 0.81 | -1.91 ± 2.96 | 0.52 |
|  | 3+ TVs | 3.74 ± 3.89 | 0.33 | -6.10 ± 5.78 | 0.29 | -1.27 ± 2.88 | 0.65 | 1.12 ± 3.38 | 0.74 |
| Site |  |  | 0.81 |  | 0.49 |  | 0.50 |  | 0.72 |
| *^Assessed using generalized mixed models; Time represents difference between late pregnancy and early pregnancy; Site p-value is from type III test of fixed effects; Ref = referent; *p<0.05* | | | | | | | | | |

| Supplementary Table 4. Associations between correlates and selected physical activity contexts across early and late pregnancy in a U.S.-based cohort conducted in 2012-2017 | | | | | | | | | | | |
| --- | --- | --- | --- | --- | --- | --- | --- | --- | --- | --- | --- |
|  |  |  |  |  |  |  | |  |  | |  |
|  |  | Leg weights | | Racket sports | | | Running | | | Swimming | |
|  |  | minutes/week | | minutes/week | | | minutes/week | | | minutes/week | |
|  |  | *β*±*SE* | p-value | *β*±*SE* | p-value | *β*±*SE* | | p-value | *β*±*SE* | | p-value |
| Time |  | -13.66 ± 2.35 | <.0001* | -0.36 ± 0.59 | 0.54 | -6.39 ± 1.74 | | 0.004* | -2.53 ± 1.48 | | 0.09 |
| Age |  | 0.20 ± 0.09 | 0.02* | 0.01 ± 0.03 | 0.62 | 0.14 ± 0.06 | | 0.02* | -0.04 ± 0.06 | | 0.54 |
| Race |  |  |  |  |  |  | |  |  | |  |
|  | White | Ref |  | Ref |  | Ref | |  | Ref | |  |
|  | Hispanic | 0.90 ± 2.94 | 0.75 | -0.24 ± 1.19 | 0.83 | 1.15 ± 2.16 | | 0.59 | -0.26 ± 2.22 | | 0.90 |
|  | Black | -1.45 ± 4.14 | 0.72 | -1.79 ± 1.65 | 0.27 | -1.54 ± 2.99 | | 0.60 | -1.28 ± 3.08 | | 0.67 |
|  | Other | 0.82 ± 4.74 | 0.86 | -2.11 ± 1.91 | 0.27 | -1.31 ± 3.44 | | 0.70 | -5.95 ± 3.54 | | 0.09 |
| Income | |  |  |  |  |  | |  |  | |  |
|  | >75,000 USD | Ref |  | Ref |  | Ref | |  | Ref | |  |
|  | 25-75,000 USD | -7.05 ± 2.98 | 0.01* | 0.53 ± 1.20 | 0.65 | 0.60 ± 2.18 | | 0.78 | -6.37 ± 2.23 | | 0.004* |
|  | <25,000 USD | -9.87 ± 4.38 | 0.03* | 1.57 ± 1.78 | 0.37 | 1.57 ± 3.21 | | 0.62 | -5.11 ± 3.30 | | 0.12 |
| Early pregnancy BMI | | -0.19 ± 0.25 | 0.43 | 0.15 ± 0.10 | 0.14 | -0.44 ± 0.18 | | 0.01* | 0.15 ± 0.19 | | 0.41 |
| Parity |  | -0.82 ± 1.40 | 0.55 | -0.59 ± 0.56 | 0.29 | -1.95 ± 1.03 | | 0.05 | -2.07 ± 1.05 | | 0.05 |
| Early pregnancy Employment | |  |  |  |  |  | |  |  | |  |
|  | Employed (day shift) | Ref |  | Ref |  | Ref | |  | Ref | |  |
|  | Employed (other shift) | 1.93 ± 3.13 | 0.53 | -1.58 ± 1.26 | 0.21 | -0.08 ± 2.28 | | 0.97 | -0.68 ± 2.34 | | 0.77 |
|  | Not employed | -1.84 ± 3.27 | 0.57 | 2.43 ± 1.32 | 0.06 | -0.25 ± 2.41 | | 0.91 | 5.59 ± 2.46 | | 0.02* |
| Early pregnancy living situation | |  |  |  |  |  | |  |  | |  |
|  | Own house/condo | Ref |  | Ref |  | Ref | |  | Ref | |  |
|  | Rent | -0.65 ± 2.86 | 0.81 | -0.54 ± 1.11 | 0.62 | -0.67 ± 2.02 | | 0.74 | 5.65 ± 2.07 | | 0.006* |
|  | Live in home of parents or other adults | 0.39 ± 3.26 | 0.90 | 2.59 ± 2.02 | 0.20 | 0.27 ± 3.67 | | 0.94 | 3.82 ± 3.75 | | 0.30 |
| TVs in the home | |  |  |  |  |  | |  |  | |  |
|  | 1 TV | Ref |  | Ref |  | Ref | |  | Ref | |  |
|  | 2 TVs | 0.13 ± 2.77 | 0.96 | -3.55 ± 1.15 | 0.002* | -1.47 ± 2.09 | | 0.48 | -1.84 ± 2.14 | | 0.38 |
|  | 3+ TVs | -2.08 ± 4.97 | 0.67 | -1.71 ± 1.30 | 0.19 | 1.39 ± 2.37 | | 0.55 | -1.08 ± 2.43 | | 0.65 |
| Site |  |  | 0.16 |  | 0.23 |  | | 0.51 |  | | 0.74 |
| *^Assessed using generalized mixed models; Time represents difference between late pregnancy and early pregnancy; Site p-value is from type III test of fixed effects; Ref = referent; *p<0.05* | | | | | | | | | | | |

| Supplementary Table 5. Associations between correlates and selected physical activity contexts, exercise days, and number of guidelines met across early and late pregnancy in a U.S.-based cohort conducted in 2012-2017 | | | | | | | | | | | | |
| --- | --- | --- | --- | --- | --- | --- | --- | --- | --- | --- | --- | --- |
|  |  | Vigorous activities (other) | | Walking | | Yoga | | Exercise days | | | Guidelines | |
|  |  | minutes/week | | minutes/week | | minutes/week | | days/week | | | range: 0-3 | |
|  |  | *β*±*SE* | p-value | *β*±*SE* | p-value | *β*±*SE* | p-value | *β*±*SE* | p-value | *β*±*SE* | | p-value |
| Time | | -0.13 ± 1.92 | 0.94 | -3.72 ± 14.72 | 0.80 | -1.23 ± 2.32 | 0.59 | -0.32 ± 0.15 | 0.03* | -0.21 ± 0.04 | | <.0001* |
| Age | | 0.02 ± 0.09 | 0.81 | 1.43 ± 0.62 | 0.02* | 0.09 ± 0.11 | 0.42 | -0.001 ± 0.007 | 0.87 | 0.001 ± 0.002 | | 0.49 |
| Race | |  |  |  |  |  |  |  |  |  | |  |
|  | White | Ref |  | Ref |  | Ref |  | Ref | . |  | |  |
|  | Black | -9.13 ± 4.39 | 0.03* | -60.00 ± 28.02 | 0.03* | -11.67 ± 5.34 | 0.02* | -0.68 ± 0.35 | 0.05 | -0.29 ± 0.13 | | 0.02* |
|  | Hispanic | -4.38 ± 3.17 | 0.16 | -18.85 ± 19.99 | 0.34 | -8.23 ± 3.82 | 0.03* | -0.59 ± 0.25 | 0.01* | 0.13 ± 0.10 | | 0.18 |
|  | Other | -3.27 ± 5.06 | 0.51 | -10.79 ± 31.64 | 0.73 | -6.26 ± 6.08 | 0.30 | -0.09 ± 0.399 | 0.81 | -0.06 ± 0.15 | | 0.67 |
| Income | |  |  |  |  |  |  |  |  |  | |  |
|  | >75,000 USD | Ref |  | Ref |  | Ref |  | Ref | . |  | |  |
|  | 25-75,000 USD | 2.41 ± 3.20 | 0.45 | -27.5 ± 20.28 | 0.17 | -7.27 ± 3.83 | 0.06 | -0.31 ± 0.25 | 0.22 | 0.09 ± 0.10 | | 0.37 |
|  | <25,000 USD | -5.81 ± 4.71 | 0.21 | -39.16 ± 30.59 | 0.20 | -6.12 ± 5.64 | 0.27 | -0.05 ± 0.38 | 0.89 | 0.18 ± 0.142 | | 0.20 |
| Early pregnancy BMI | | -0.06 ± 0.27 | 0.80 | -2.88 ± 1.67 | 0.08 | -0.05 ± 0.32 | 0.86 | -0.02 ± 0.02 | 0.16 | -0.01 ± 0.007 | | 0.04* |
| Parity | | 0.84 ± 1.49 | 0.57 | -3.31 ± 9.74 | 0.73 | -5.50 ± 1.80 | 0.002* | -0.18 ± 0.12 | 0.13 | 0.06 ± 0.03 | | 0.09 |
| Early pregnancy Employment | |  |  |  |  |  |  |  |  |  | |  |
|  | Employed (day shift) | Ref |  | Ref |  | Ref |  | Ref | . |  | |  |
|  | Employed (other shift) | 2.31 ± 3.34 | 0.48 | -6.36 ± 21.04 | 0.76 | 2.98 ± 4.03 | 0.46 | -0.63 ± 0.26 | 0.01* | -0.01 ± 0.09 | | 0.90 |
|  | Not employed | -0.77 ± 3.53 | 0.82 | 10.73 ± 22.37 | 0.63 | 4.24 ± 4.19 | 0.31 | 0.47 ± 0.28 | 0.09 | -0.12 ± 0.09 | | 0.18 |
| Early pregnancy living situation | |  |  |  |  |  |  |  |  |  | |  |
|  | Own house/condo | Ref |  | Ref |  | Ref |  | Ref | . |  | |  |
|  | Rent | -1.17 ± 2.96 | 0.69 | 12.42 ± 18.45 | 0.50 | -1.26 ± 3.57 | 0.72 | 0.04 ± 0.23 | 0.85 | 0.07 ± 0.08 | | 0.39 |
|  | Live in home of parents or other adults | -1.49 ± 5.43 | 0.77 | 13.09 ± 34.02 | 0.70 | 0.03 ± 6.40 | 0.99 | -0.19 ± 0.42 | 0.65 | 0.21 ± 0.13 | | 0.11 |
| TVs in the home | |  |  |  |  |  |  |  |  |  | |  |
|  | 1 TV | Ref |  | Ref |  | Ref |  | Ref | . |  | |  |
|  | 2 TVs | -0.02 ± 3.06 | 0.99 | -16.43 ± 19.29 | 0.39 | 3.36 ± 3.69 | 0.36 | -0.03 ± 0.24 | 0.88 | -0.0 ± 0.09 | | 0.83 |
|  | 3+ TVs | -2.96 ± 3.47 | 0.39 | -3.95 ± 21.58 | 0.85 | 0.22 ± 4.17 | 0.95 | -0.22 ± 0.27 | 0.39 | -0.02 ± 0.09 | | 0.78 |
| Site |  |  | 0.06 |  | 0.004* |  | 0.02* |  | 0.21 |  | | 0.65 |
| *^Assessed using generalized mixed models; Time represents difference between late pregnancy and early pregnancy; Site p-value is from type III test of fixed effects; = referent; *p<0.05* | | | | | | | | | | | | |

| Supplementary Table 6. Associations between correlates and sedentary behavior context across early and late pregnancy in a U.S.-based cohort conducted in 2012-2017 | | | | | | | | | | | |
| --- | --- | --- | --- | --- | --- | --- | --- | --- | --- | --- | --- |
|  |  |  |  |  |  |  |  |  |  |  |  |
|  |  | Sitting - Away from home | | Sitting - TV/Screens | | Sitting - Other home | | Standing- Away from home | | Standing - Home | |
|  |  | hours/week | | hours/week | | hours/week | | hours/week | | hours/week | |
|  |  | *β*±*SE* | p-value | *β*±*SE* | p-value | *β*±*SE* | p-value | *β*±*SE* | p-value |  |  |
| Time | | -0.30 ± 0.89 | 0.73 | 0.49 ± 0.74 | 0.50 | -0.16 ± 0.69 | 0.81 | -2.76 ± 0.87 | 0.001* | -0.32 ± 2.82 | 0.90 |
| Age | | -0.02 ± 0.05 | 0.58 | -0.03 ± 0.03 | 0.37 | -0.06 ± 0.03 | 0.04* | -0.02 ± 0.05 | 0.61 | -0.13 ± 0.15 | 0.36 |
| Race | |  |  |  |  |  |  |  |  |  |  |
|  | White | Ref |  | Ref |  | Ref |  | Ref |  | Ref |  |
|  | Black | -3.07 ± 2.54 | 0.22 | -0.15 ± 1.79 | 0.93 | 1.23 ± 1.52 | 0.41 | -0.59 ± 2.63 | 0.82 | -18.0 ± 7.34 | 0.007* |
|  | Hispanic | -4.57 ± 2.00 | 0.02* | -3.86 ± 1.40 | 0.006* | -3.18 ± 1.19 | 0.008* | -0.04 ± 2.07 | 0.98 | -15.3 ± 5.76 | 0.01* |
|  | Other | -1.26 ± 2.91 | 0.66 | -1.95 ± 2.03 | 0.33 | 0.16 ± 1.73 | 0.92 | -1.03 ± 3.01 | 0.73 | -4.45 ± 8.36 | 0.59 |
| Income | |  |  |  |  |  |  |  |  |  |  |
|  | >75,000 USD | Ref |  | Ref |  | Ref |  | Ref |  | Ref |  |
|  | 25-75,000 USD | -4.23 ± 1.96 | 0.03* | -1.29 ± 1.37 | 0.34 | -1.40 ± 1.16 | 0.22 | 3.760 ± 2.03 | 0.06 | 9.92 ± 5.64 | 0.07 |
|  | <25,000 USD | -5.58 ± 2.74 | 0.04* | -1.41 ± 1.92 | 0.46 | -3.09 ± 1.64 | 0.05 | -1.18 ± 2.84 | 0.67 | -0.20 ± 7.90 | 0.97 |
| Early pregnancy BMI | | 0.12 ± 0.14 | 0.37 | 0.25 ± 0.09 | 0.01* | 0.16 ± 0.08 | 0.05 | 0.15 ± 0.14 | 0.29 | 0.06 ± 0.40 | 0.86 |
| Parity | | -1.17 ± 0.73 | 0.11 | -0.76 ± 0.51 | 0.14 | -0.39 ± 0.44 | 0.36 | 0.52 ± 0.76 | 0.49 | 3.55 ± 2.12 | 0.09 |
| Early pregnancy Employment | |  |  |  |  |  |  |  |  |  |  |
|  | Employed (day shift) | Ref |  | Ref |  | Ref |  | Ref |  | Ref |  |
|  | Employed (other shift) | -6.05 ± 1.76 | 0.001* | 0.62 ± 1.24 | 0.61 | 1.49 ± 1.05 | 0.15 | 4.70 ± 1.83 | 0.01* | 8.76 ± 5.09 | 0.08 |
|  | Not employed | -8.08 ± 1.75 | <.001* | 2.01 ± 1.22 | 0.10 | 1.44 ± 1.04 | 0.16 | -4.55 ± 1.81 | 0.01* | 12.80 ± 5.04 | 0.01* |
| Early pregnancy living situation | |  |  |  |  |  |  |  |  |  |  |
|  | Own house/condo | Ref |  | Ref |  | Ref |  | Ref |  | Ref |  |
|  | Rent | -4.34 ± 1.70 | 0.01* | 0.92 ± 1.19 | 0.44 | -0.32 ± 1.01 | 0.75 | 0.93 ± 1.76 | 0.59 | -1.06 ± 4.91 | 0.82 |
|  | Live in home of parents or other adults | -4.06 ± 2.57 | 0.11 | 2.92 ± 1.80 | 0.10 | 1.50 ± 1.53 | 0.32 | 3.99 ± 2.66 | 0.13 | 1.64 ± 7.39 | 0.82 |
| TVs in the home | |  |  |  |  |  |  |  |  |  |  |
|  | 1 TV | Ref |  | Ref |  | Ref |  | Ref |  | Ref |  |
|  | 2 TVs | 2.25 ± 1.72 | 0.19 | 0.49 ± 1.21 | 0.68 | -1.42 ± 1.02 | 0.16 | -0.38 ± 1.78 | 0.83 | 0.79 ± 4.96 | 0.87 |
|  | 3+ TVs | 2.50 ± 1.79 | 0.16 | 0.27 ± 1.26 | 0.83 | -0.30 ± 1.07 | 0.77 | -2.78 ± 1.86 | 0.13 | -2.25 ± 5.17 | 0.82 |
| Site |  |  | 0.79 |  | 0.58 |  | 0.70 |  | 0.01* |  | 0.006* |
| *^Assessed using generalized mixed models; Time represents difference between late pregnancy and early pregnancy; Site p-value is from type III test of fixed effects; Ref = referent; *p<0.05* | | | | | | | | | | | |
